# *Bifidobacterium adolescentis* (DSM 20083) and *Lactobacillus casei* (Lafti L26-DSL): Probiotics Able to Block the In Vitro Adherence of Rotavirus in MA104 Cells

**DOI:** 10.1007/s12602-017-9277-7

**Published:** 2017-04-21

**Authors:** Karem Prunella Fernandez-Duarte, Nury Nathalia Olaya-Galán, Sandra Patricia Salas-Cárdenas, Jazmin Lopez-Rozo, Maria Fernanda Gutierrez-Fernandez

**Affiliations:** Infectious Diseases Research Group, Department of Microbiology, Pontificia Universidad Javeriana, Carrera 7 #40-62, (50-123), Bogota, Colombia

**Keywords:** Rotavirus, Antiviral effect, Protein-based metabolites, *Lactobacillus casei*, *Bifidobacterium adolescentis*, Co-incubation

## Abstract

Rotavirus is the leading worldwide cause of gastroenteritis in children under five years of age. Even though there are some available vaccines to prevent the disease, there are limited strategies for challenging diarrhea induced by rotavirus infection. For this reason, researchers are constantly searching for other approaches to control diarrhea by means of probiotics. In order to demonstrate the ability of some probiotic bacteria to interfere with the *in vitro* rotavirus infection in MA104 cells, strains of *Lactobacillus* sp. and *Bifidobacterium* sp. were tested in MA104 cells before the viral infection. As a preliminary assay, a blocking effect treatment was performed with viable bacteria. In this screening assay, four of initial ten bacteria showed a slight reduction of the viral infection (measured by percentage of infection). *L. casei* (Lafti L26-DSL), *L. fermentum*(ATCC 9338), *B. adolescentis* (DSM 20083), and *B. bifidum* (ATCC 11863) were used in further experiments. Three different treatments were tested in order to evaluate protein-based metabolites obtained from mentioned bacteria: (i) cell exposure to the protein-based metabolites before viral infection, (ii) exposure to protein-based metabolites after viral infection, and (iii) co-incubation of the virus and protein-based metabolites before viral infection to the cell culture. The best effect performed by protein-based metabolites was observed during the co-incubation assay of the virus and protein-based metabolites before adding them into the cell culture. The results showed 25 and 37% of infection in the presence of *L. casei* and *B. adolescentis* respectively. These results suggest that the antiviral effect may be occurring directly with the viral particle instead of making a blocking effect of the cellular receptors that are needed for the viral entrance.

## Background

Worldwide, acute diarrheal disease (ADD) remains as one of the most common diseases affecting people of all ages, but its frequency and severity are higher in children under the age of 5 [1]. About 600,000 children die every year as a consequence of rotavirus infection, with more than 80% of all rotavirus-related deaths occurring in low-income countries in south Asia and sub-Saharan Africa [2]. Globally, rotavirus-related deaths represent approximately 5% of all deaths in children of this age. However, in other regions of developing countries, mortality is not so high but important rates of morbidity still remain in spite of the availability of a polyvalent vaccine worldwide [3].

Probiotics are defined as “lived microorganisms that, when administered in adequate amounts, confer a health benefit on the host” [4, 5]. Some of these benefits have been established on human health in the infectious disease field: (i) interaction and enhancement of the immune system, (ii) production of antimicrobial substances, (iii) enhancement of the mucosal barrier function, and (iv) competition with enteropathogens [6–8]. Some studies have demonstrated the beneficial effect of probiotics against rotavirus infection [9, 10]. Most of them are clinical assays proving that the use of probiotics can lessen severity and duration of rotavirus diarrhea [11], whereas other studies are performed in vitro directed to the understanding of molecular and biochemical pathways associated with the mechanism employed by probiotics to accomplish the antiviral activity [12–14].

Even if most of these studies show the effectiveness of probiotics or their metabolic products on viral multiplication (both clinical and in vitro assays), some strategies have been established associated to the antiviral effect such as blocking effect, intracellular regulation, and immune response modulation [15–17]; however, evidence is not enough to clarify the mechanisms by which these processes occur, giving way to further studies in order to understand better the antiviral effect mediated by probiotic bacteria.

As a manner of fact, so as to advance the knowledge related to the strategies used by probiotics against viruses, we proposed as a hypothesis in which the probiotic bacteria were able to block in vitro rotavirus infection by altering the process of adhesion of the virus onto the cells. The aim of this study was to determine if the antiviral effect of probiotics was given by the competition of receptors on culture cells and whether this effect was caused by the whole and viable bacteria and/or was due to its protein-based metabolites.

## Materials and Methods

### Cell Lines and Virus

The MA-104 cell line (embryonic Rhesus monkey kidney cells) were grown in advanced DMEM supplemented with 4% fetal calf serum (Gibo, Invitrogen), l-glutamine (2 mmol/L), antibiotic, and antimycotic, at 37 °C in 5% CO_2_ atmosphere in tissue culture flasks until confluency. The cell culture medium was regularly changed. For the assays, 150,000 cells per well were placed in 24-well plates and incubated at the same conditions. After 24 h and 90% of confluence, each well contained about 500,000 cells.

Rotavirus RRV strain (Rhesus monkey) was kindly donated by Dr. Carlos Guerrero of the Universidad Nacional de Colombia. The infection was performed with a MOI of 5 and the virus was previously activated with trypsin (10 μg/mL). The MOI of 5 was used in order to saturate the MA104 cell culture, simulating what could occur during a natural viral infection in mature enterocytes, where the amount of virus is greater than the amount of cells.

### Probiotic Bacteria and Growth Conditions

*L. casei* (Lafti L26-DSL), *L. rhamnosus* (ATCC 7469), *L. fermentum* (ATCC 9338), *L. plantarum* (CECT 220), *L. acidophilus* (Lafti L10-DSL), *B. lactis* (Lafti B94-DSL), *B. breve* (ATCC 15700), *B. adolescentis* (DSM 20083), *B. bifidum* (ATCC 11863), and *B. dentium* (DSM 20084) were activated in MRS broth at 37 °C under anaerobic conditions and stored at −80 °C in the same broth supplemented with 25% glycerol (*v*/*v*) and skim milk 10% (*w*/*v*). The number of viable bacteria in 1 mL of bacterial culture was determined by measuring optical density at 580 nm. The number was determined by extrapolation of the internal laboratory standard curve. For experimental approaches, bacteria cultures were centrifuged at 3000×*g* for 10 min and bacteria were resuspended in DMEM for all the antiviral assays. The final bacterial suspension contained 1 × 10^8^ CFU/mL as previously reported [18].

Bacteria were maintained under anaerobic conditions by streaking method in MRS solid culture media with AnaeroGen sachets (OXOID) in anaerobic jars. For experiments, bacteria were grown in MRS broth under anaerobic conditions as previously described by Hungate in 1969 [19]. Briefly, MRS broth was heated until boiling, and then gas exchange was performed with a constant flux of nitrogen (N_2_) gas. A final gas exchange was performed with a mixture of 80:20 of nitrogen (N_2_) and carbon dioxide (CO_2_) in order to remove remaining oxygen present in the media.

### Recovery of Bacteria Protein-Based Metabolites

Bacterial culture supernatants were obtained from growing bacterial cultures in 250 mL MRS broth under anaerobic conditions until the exponential growth of each bacterium was accomplished (recollection times differ between 8 and 10 h of bacterial growth) at 37 °C. Bacteria were removed by centrifugation at 3000×*g* for 10 min. Supernatants were recovered and filtered through a pore of 0.22 μm. Protein-based metabolites were precipitated with 10% PEG (*w*/*v*) overnight. After that, concentric centrifugations at 16,000×*g*, 4 °C for 30 min were performed with the aim of concentrating the protein-based metabolites present in the supernatants. Proteins were resuspended in 2.5 mL of sterile PBS and stored at −20 °C until use. The proteins were quantified using the BCA kit Thermo Scientific. For both cytotoxicity and antiviral assays, a free bacteria broth control was considered, which was also precipitated with PEG and compared with metabolites for viability and with the positive control in the antiviral assays.

### Cytotoxicity Assays

To perform biological assays, MA-104 cells were separately seeded in 96-well plates until confluence. Cytotoxic effect was tested for each probiotic bacterium and protein-based metabolites by tenfold serial dilutions added to the confluent cells. Cells exposed to bacteria or protein-based metabolites were incubated for 90 min at 37 °C. After that, cells were washed twice with PBS and were incubated for 24 h at 37 °C with 5% CO_2_. Cytotoxicity effect was determined by 0.4% trypan blue visualized in a light microscope for viable bacteria. For protein-based metabolites, cytotoxicity was tested using MTT salts (SIGMA-Aldrich, Saint Louis), in order to determine the formation of formazan products detected in a Multiskan MCC/340 (Thermo Fisher Scientific, Waltham) spectrophotometer at a wavelength of 540 nm. Cytotoxicity assays for whole and viable bacteria were tested at concentrations between 10^6^ and 10^8^ FCU/mL as reported by Botic et al. [18]. Protein-based metabolites were tested in ranges between 10 and 1000 μg/mL diluted in DMEM as well as pure metabolite.

### Antiviral Assays

*Inhibition of viral infection by the whole and viable bacteria*: Ten probiotic bacteria [20] were tested against RV infection by the principle of blocking the viral entrance to the cells. For these experiments, cells were first incubated with viable probiotic bacteria (500 μL, 10^8^ CFU/mL) for 90 min at 37 °C and 5% CO_2_. After incubation, cell cultures were washed to remove unattached bacteria from the cells with DMEM without supplements and monolayers were challenged with RV infection for 10 h at 37 °C and 5% CO_2_. Percentage of viral infection was measured by flow cytometry. These experiments were the preliminary tests to select the bacteria with major activity with the aim of using them in further assays.

*Inhibition of viral infection by bacterial protein-based metabolites* (*pre-treatment*): Cells were first incubated with 100 μg/mL of each metabolite for 90 min. After this time, the unbounded protein-based metabolites were washed out from the cells with DMEM without supplements and monolayers were challenged with RV infection for 10 h at 37 °C, 5% CO_2_.

*Inhibition of viral infection due to a co-incubation assay*: Bacterial protein-based metabolites were first co-incubated with RV (previously activated with 10 μg/mL trypsin) in DMEM for 60 min at 37 °C and 5% CO_2_. After this time, the mixture was placed in contact with the MA104 cells for 1 h at 37 °C and 5% CO_2_, and then the excess of inoculum was washed and assays were incubated until 10 h of post-infection.

*Intracellular effect of the protein-based metabolites of probiotic bacteria against viral infection* (*post-infection assay*): MA104 cells were first infected with the virus as previously described, and after removing the viral inoculum, cells were washed with PBS and were exposed to bacterial protein-based metabolites (100 μg/mL) per 1 h. Unbounded protein-based metabolites were washed, and cells were incubated until 10 h as mentioned before.

For all the antiviral assays, positive and negative controls were included. Positive controls were MA104 cells infected with RRV at a MOI of 5 without any treatment. Negative controls were MA104 cells grown simultaneously in the experiments with DMEM without supplements in the culture. Antiviral assays were performed by three independent assays and duplicate each.

### Flow Cytometry: Detection of Viral Infection

The viral growth was determined by flow cytometry in all the antiviral assays. Cells were dissociated with trypsin, placed in 1.5-mL conical tubes, centrifuged at 3000×*g*, and resuspended in 500 μL sterile PBS-EDTA [21]. Cells were fixed for 15 min with 2% paraformaldehyde. After removing this paraformaldehyde, cells were washed with PBS and permeabilized with Triton X-100 0.3%. For intracellular viral detection, an anti-TLP polyclonal antibody produced in rabbit, with a titer of 1/3000 (kindly donated by Dr. Carlos Guerrero, Universidad Nacional), directed to VP6 proteins of the virus, was used. Then, as a secondary antibody, an anti-Rabbit IgG Alexa Fluor 488 (Invitrogen) diluted 1/2500 was used; staining was performed at room temperature in dark conditions. Cells were washed twice with PBS and were resuspended in FACS-flow until analysis. A FACS Aria II cytometer (Becton Dickinson) was used for analysis, where percentages of positives cells were included. Further analyses were performed with Flow-Jo software.

### Statistical Analysis

ANOVA and Dunnett’s tests were used as a parametric statistical analysis in order to find if the percentage of infected cells and the presence of the viral antigen inside the cells treated with probiotics or its protein-based metabolites were significant compared with the positive control (*p* < 0.05).

## Results

### Cytotoxicity Tests

Cytotoxicity tests were performed to determine the maximum concentration at which bacteria or protein-based metabolites can be used on MA104 cell line. For whole and viable bacteria, it was found that a concentration of 1 × 10^8^ CFU/mL of bacteria did not show toxicity over the MA104 cell line, shown by the trypan blue stain, where cell viability was higher than 90% in all the cases. Viable bacteria results show that the ten probiotic strains were not toxic for MA104 cell line and could be used in further experiments.

**Fig. 1:**
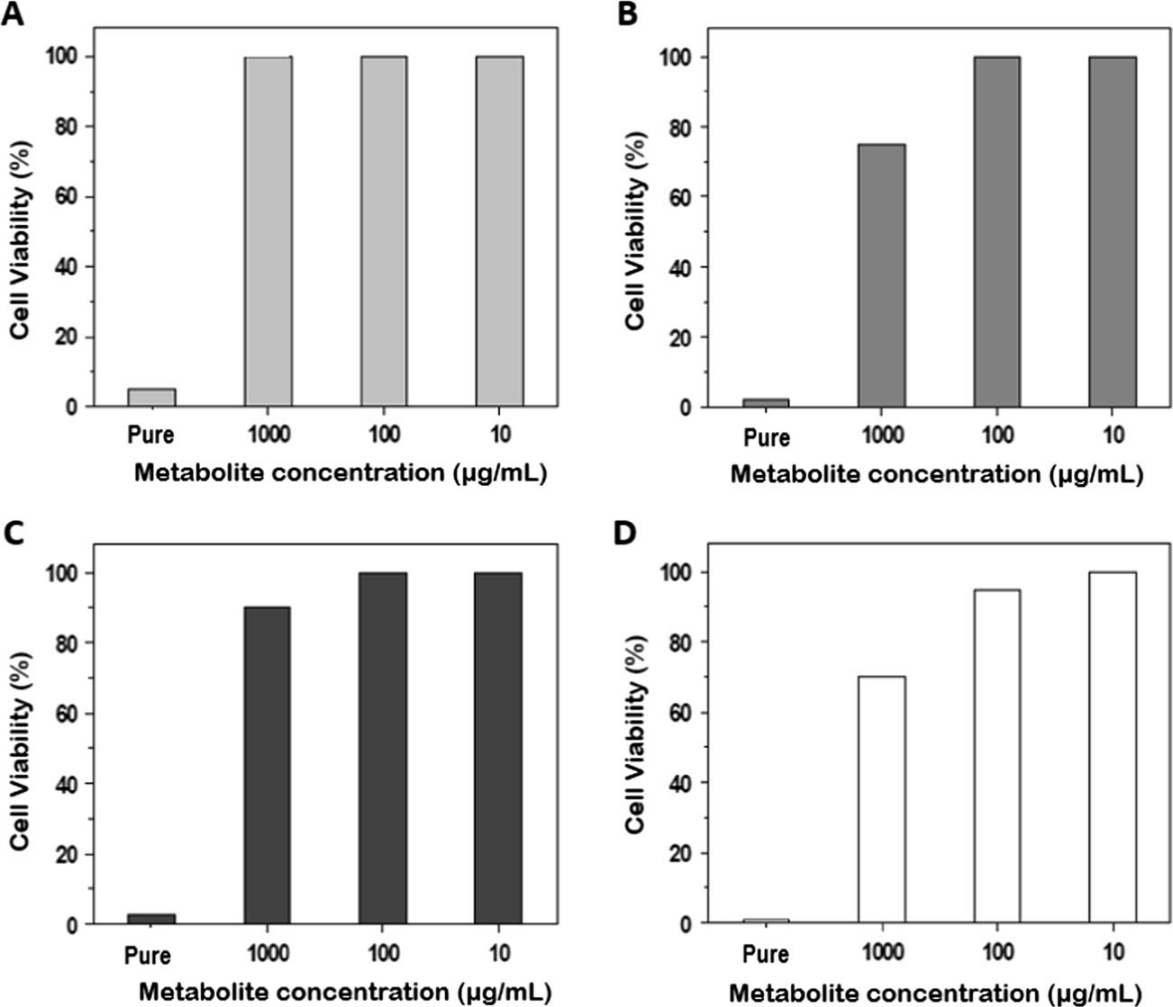
Cytotoxicity effect of bacterial metabolites tested in MA104 cells. **a** *L. casei*. **b** *L. fermentum*. **c** *B. adolescentis*. **d** *B. bifidum*. Notice 100% of cell viability when using 100 μg/mL with the metabolites of the four bacteria

In the case of the bacterial protein-based metabolites, folded dilutions in DMEM were analyzed with MTT technique, where in general, a concentration of 100 μg/mL for all the metabolites was atoxic for the MA104 cell culture. Viability was obtained between 90 and 100% in the presence of the four metabolites. In contrast, when testing pure metabolites (without dilutions in DMEM), high toxic effects were obtained (Fig. 1).

### Preliminary Screening of Bacteria with Potential Antiviral Activity

**Fig. 2:**
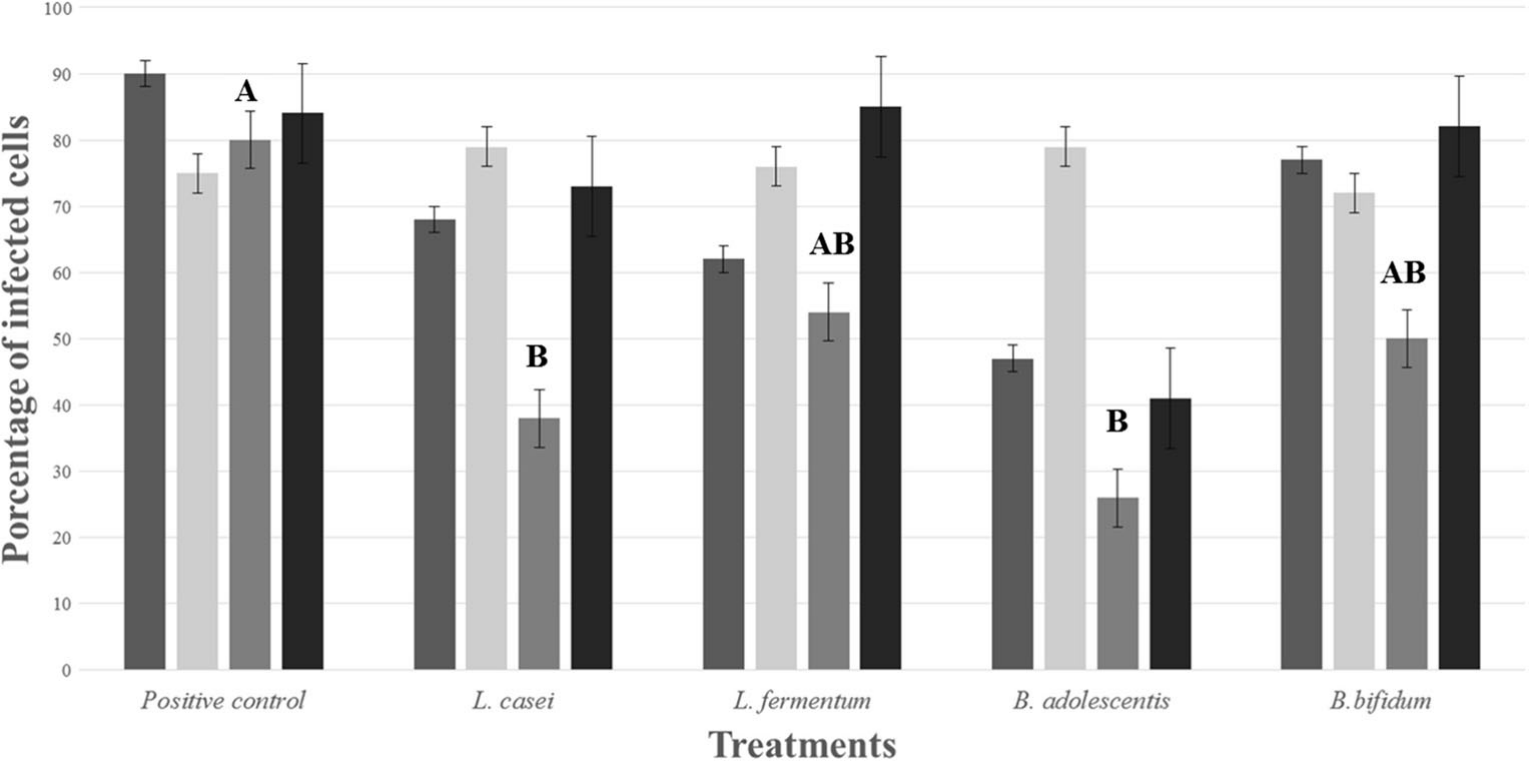
Effect of *L. casei*, *L. fermentum*, *B. adolescentis*, and *B. bifidum* and its metabolite products on RV infection in MA104 cells. (*dark gray*) Screening assay: viable bacteria, blocking effect. Evaluation of antiviral treatments: protein-based metabolites, (*light gray*) pre-treatment; (*gray*) co-incubation; (*black*) post-infection. Statistical analysis showed significant results in the co-incubation treatment when compared with positive control (*A*) (*letters AB; p = 0.005;* *letter B*; *p =* 0.001)

Growth conditions of the ten probiotic strains are shown in Table 1. In the preliminary assay against RV infection, non-significant results were obtained; however, the 10 tested bacteria, *L. casei*, *L. fermentum*, *B. adolescentis*, and *B. bifidum*, were the ones with highest antiviral activity by a tentative blocking effect of the viral entrance. A reduction of the viral infection of 31, 37, 42, and 24%, respectively, measured by flow cytometry, was obtained. In contrast, viral infection in the positive control (MA104 cells infected with the virus) was around of 90% of positive cells. These four bacterial strains were selected to continue further inhibition experiments using primary protein-based metabolites derived from their growth (Fig. 2, viable bacteria).

**Table 1:** Conditions of bacteria cultures during exponential phase and preliminary antiviral assays

Probiotic strains	Exponential growth conditions			Preliminary assay
	Time (hours)	OD (580 nm)	Log_10_ CFU/mL	Percentage of viral infection^a^
*L. casei* (Lafti L26-DSL)	8	0.43	7.94	69%
*L. rhamnosus (*ATCC 7469)	8	0.289	7.43	80%
*L. fermentum (*ATCC 9338)	8	0.47	6.67	63%
*L. plantarum* (CECT 220)	8	0.403	8.31	76%
*L. acidophilus* (Lafti L10-DSL)	10	0.202	6.94	78%
*B. lactis* (Lafti B94-DSL)	10	0.09	6.23	75%
*B. breve* (ATCC 15700)	6	0.1	6.83	72%
*B. adolescentis* (DSM 20083)	8	0.054	6.68	58%
*B. bifidum* (ATCC 11863)	6	0.151	4.82	67%
*B. dentium* (DSM 20084)	8	0.6	7.86	80%

^a^Positive control for preliminary assays had a 90% of infected cells with RRV

After selection of the four strains with the best antiviral effect, the metabolites of each bacteria were recovered. After precipitation with PEG, quantification was performed by BCA technique. Each metabolite was obtained in three independent culture batches for the antiviral assays. Results of the amount of protein could be seen in Table 2.

**Table 2:** Protein-based metabolites quantification after precipitation with PEG

	Probiotic metabolites concentrations (μg/mL)			
Batch	*L. casei*	*L. fermentum*	*B. adolescentis*	*B. bifidum*
1	1001.33	742.17	1040.22	758.56
2	706.06	1680.78	694.94	1540.22
3	1214.94	1376.61	556.06	1716.06

Data is shown as mean of three independent measures for each bacteria/batch

### Antiviral Activity of Protein-Based Metabolites

#### Inhibition of Viral Infection by Bacterial Protein-Based Metabolites: Pre-Treatment Assay

Pre-treatment period of 90 min on MA104 cells with probiotic protein-based metabolites followed by viral infection did not show any differences between the treatments and the positive control. Percentage of infection in the treatments was between 70 and 75%, which was the same as that in the positive control (71%). These results suggest that the protein-based metabolites were not significant in performing a blocking effect of cellular receptors in the viral infection (Fig. 2, pre-treatment).

#### Reduction of the Viral Infectivity: Co-Incubation Assay between Virus and Protein-Based Metabolites

To determine whether probiotic protein-based metabolites were able to interact directly with the virus, and thus affect the viral attachment to the cells, the co-incubation assay was performed. The results showed a significant decrease in the viral infection. The percentage of infected cells in the presence of the *B. adolescentis* (26%) decreased markedly in comparison with the positive control (80%). A similar behavior was found in the presence of *L. casei*, where the percentage of infected cells was significantly reduced to a 38%. Likewise, the protein-based metabolites of the other bacteria also showed a significant result (*P* < 0.05) decreasing the percentage of infected cells to 54 and 50% (Fig. 2, co-incubation).

#### Effect of Protein-Based Metabolites after Viral Infection

In the last strategy analyzed, it could be observed that the single metabolite that achieved a significant reduction in the viral infection was the one obtained from *B. adolescentis* with a *P* value =0.001. The other three protein-based metabolites did not show any activity by this assay (Fig. 2, post-treatment).

## Discussion

Taking into account that rotavirus infection is still one of the most important diseases in developing countries that affect children under the age of five [22, 23], all the possible alternatives directed to improve the life quality of children should be considered. Thus, the use of probiotics to counteract the effect of rotavirus in the human population arises as an important strategy to manage the disease.

Although probiotics have been reported in several studies against rotavirus infection [13, 14, 17, 18], the specific mechanism by which the antiviral effect is mediated remains unclear. Even if probiotics are also used in the prevention and therapy of diarrhea, non-standardized conditions have been established in clinical trials, as well as the definition of the probiotic strains with best results [11].

Particularly in this study, the objective was to determine whether probiotics or their protein-based metabolites had the ability to interfere with the first steps of the viral cycle, which are viral adhesion or penetration, being fundamental steps for the viral infection [24]. The first approach of this work was evaluated with whole and viable bacteria in an early stage of the viral cycle. It was expected that viable bacteria attached to the cell surface in order to colonize the MA104 cell monolayers and thus cellular receptors involved in in vitro adhesion [18]. In the preliminary results of the screening assays, a blockage of the viral entrance was observed and could agree with the proposal of some other studies regarding the antiviral effect of probiotics [25–29].

Even if specific receptors were not tested, these preliminary results showed a reduction in the viral infection measured by flow cytometry in four out of the ten probiotic strains tested. One of these four bacteria was *B. adolescentis*, which has been previously reported as a potential microorganism with an antiviral activity against different viruses [14, 30–32].

Now, in spite of the wide use of probiotics in many fields, there are few cases where the use of viable bacteria could lead to opportunistic infections, allergic reactions, or autoimmune responses [33]. This is the reason why the use of probiotic-derived products has arisen in a new field denominated metabiotics [34]. Experimental approaches tested with these probiotic-derived products have also shown interesting results [35].

In this study, the probiotic strains chosen from the screening assay were *L. casei*, *L. fermentun*, *B. adolescentis*, and *B. bifidum*, where protein-based metabolic products were tested in further experiments. From the three different strategies evaluated (pre-treatment, co-incubation, and post-infection), the co-incubation assay of viral particles and protein-based metabolites showed the best results in the antiviral activity approach in comparison with the other treatments. It prevented the viral adhesion and/or penetration into the MA104 cells maybe because of a direct interaction of the protein-based metabolites with the external viral proteins such as VP7 or VP4. It is important to take into account that all experiments were performed with trypsin-activated viral particles, a fundamental process for a cleavage step of the viral proteins necessary for viral entrance to the host cell [36].

On the other hand, in the case of pre-treatment and post-infection assays, it was expected that the antiviral activity was mediated by a mechanism directed to the cells instead of affecting the viral particle. The hypothesis is that direct interactions with the cellular receptors or intracellular regulating processes could be happening. Here, in the first case, according to the suggested mechanism of probiotics [18, 37], it was expected that the interaction with cellular receptors of protein-based metabolites could block the attachment of the virus to the cell surface, and thus, the viral entrance could not be performed. In the second case, it was proposed to produce an antiviral activity associated with intracellular regulation as it was previously reported for another strain of *B. adolescentis* [38].

With this study, it could be said that protein-based metabolites obtained from *L. casei* and *B. adolescentis* were able to block rotavirus entrance by a direct effect on the viral particle, in contrast to the proposed hypothesis. It is possible that adhesion process to the MA104 cell receptors could not be efficiently performed due to an alteration in the external viral proteins; results were observed in comparison with the positive control.

Hence, results obtained in this study are a preliminary approach in order to continue analyzing the possible mechanism exerted by probiotic bacteria. An important fact to take into account in further studies is to evaluate if it could be a dose-dependent activity of probiotic metabolites, taking into account that in this study, we used high concentrations of the metabolites. Therefore, an optimization of the process could be obtained if lower concentrations are also able to perform antiviral activity.

These results contribute to strengthening the knowledge that supports the activity of probiotic bacteria against gastrointestinal viral infections. Several mechanisms have been proposed, but co-infection strategy could be considered as a novel approach against rotavirus. This strategy could be a possible alternative directed to crops that could be contaminated with RV on field. Further studies are needed to completely understand the specific mechanisms involved in the antiviral activity of probiotics; in vivo and clinical approaches are necessary in order to verify its antiviral activity. In the future, it could be proposed a simple and inexpensive biological product, with potential antiviral activity dispensed as a dietary supplement or maybe in a drug formulation.
